# Supplementary material for: Exploring Extracellular Vesicle Surface Protein Markers Produced by Glioblastoma Tumors: A Characterization Study Using In Vitro 3D Patient-Derived Cultures
Source: Cancers (Basel). 2024 Nov 6;16(22):3748. doi: 10.3390/cancers16223748 (PMC11592176; doi:10.3390/cancers16223748)
Supplement: Supplementary file 1 [file cancers-16-03748-s001.zip › cancers-3257896-supplementary.pdf]

595 nm Abs Values

	GB67	GB78	GB79	GB88	GB89	GB103	GB104	GB108	GB110
**Abs 1**	0.6063	0.4897	0.5266	0.6634	0.5242	0.4221	0.5228	0.4191	0.4724
**Abs 2**	0.6687	0.4409	0.4687	0.6820	0.5457	0.3945	0.4720	0.4862	0.4753
**Abs 3**	0.7063	0.4389	0.4888	0.6581	0.5008	0.3616	0.3858	0.4245	0.4858
**AVG**	0.6875	0.4399	0.4787	0.6700	0.5233	0.3781	0.4289	0.4553	0.4806

BSA Std Curve 595 nm Abs Values

Assay	Abs
**0**	0.3237
**0.005**	0.3335
**0.05**	0.3652
**0.5**	0.5408
**1**	0.8026
**2**	1.1975
**0**	0.3237

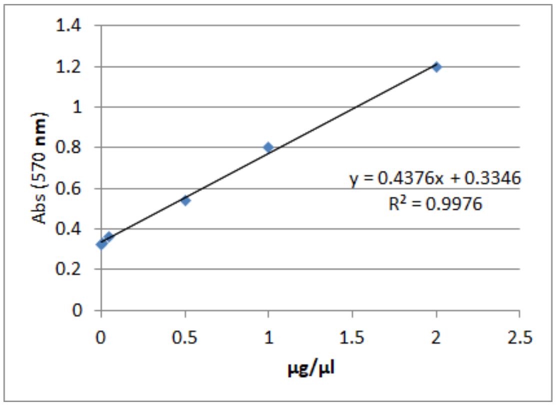

Standard Curve Equation Values

equation on graph (y = mx+B)

**Slope (m)**	**0.3437**
**X-intercept (b)**	**0.3254**

Sample Concentration (µg/µL)

GB67	GB78	GB79	GB88	GB89	GB103	GB104	GB108	GB110
1.05	0.33	0.45	1.00	0.58	0.15	0.30	0.38	0.45

Supplementary Figure S1. Protein concentration determination by Bradford assay**.**
Absorbance measurements of protein content for each sample, recorded at 595 nm and measured in triplicate. A standard curve was generated using known concentrations of bovine serum albumin (BSA) to determine the total protein concentration in each sample. The calculated protein concentrations are expressed in µg/µL.

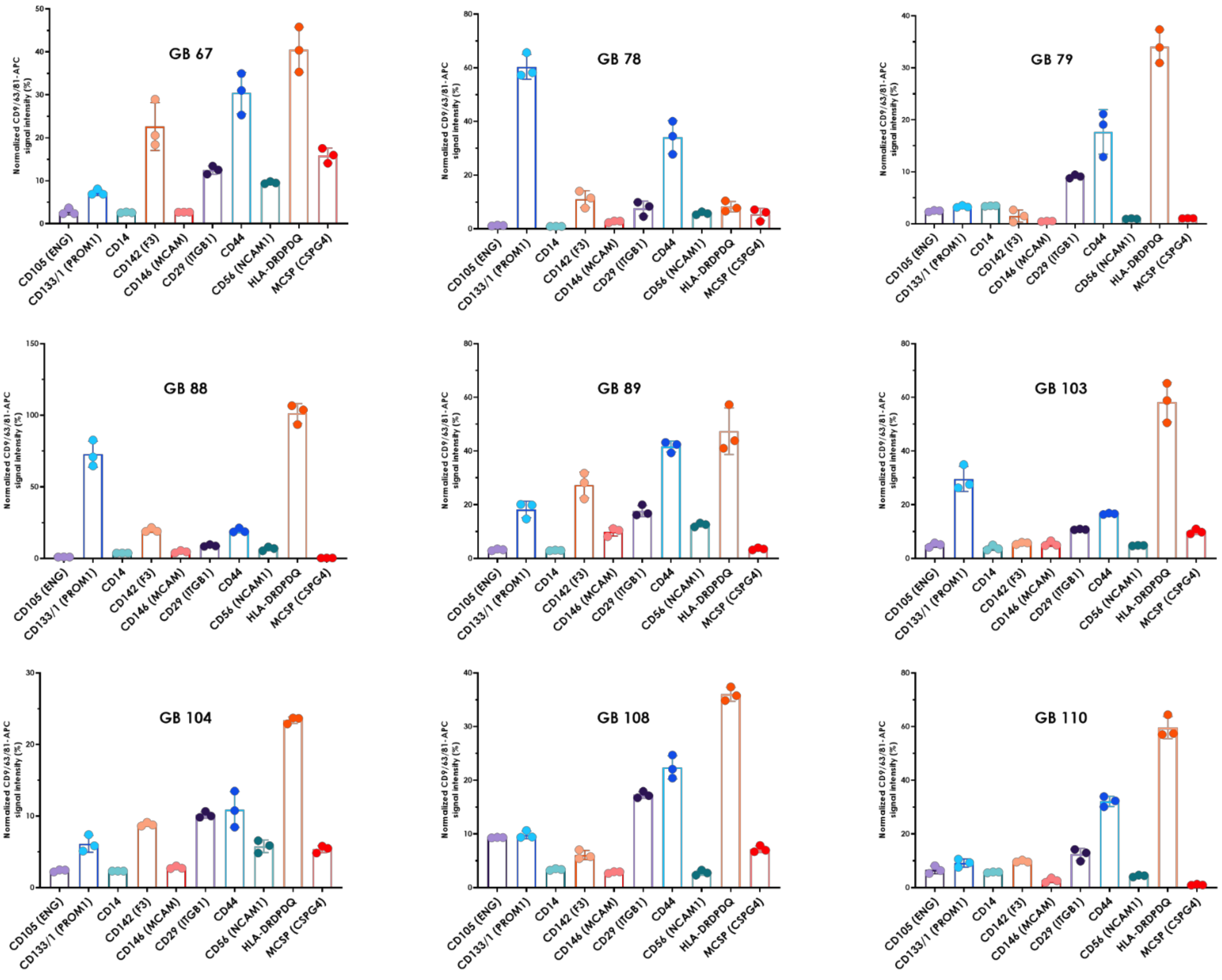

Supplementary Figure S2. Flow cytometry analysis of EVs membrane marker expression. Expression profiles of the 10 proteins, out of the 37 analyzed, that exhibited positive signals—defined as mean fluorescence intensity (MFI) levels higher than their respective isotype controls—across all tumor samples analyzed.

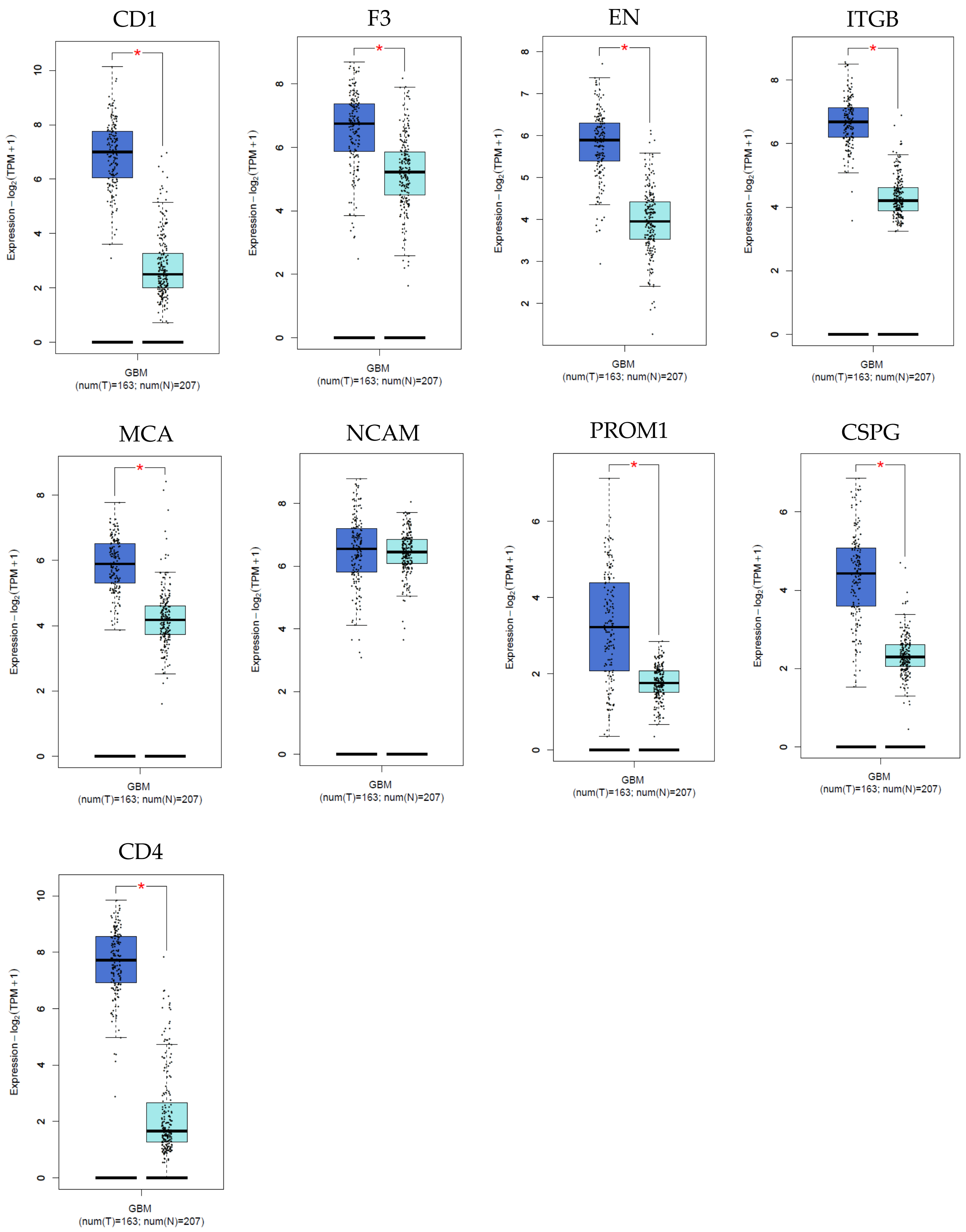

Supplementary Figure S3. Expression of EVs membrane biomarkers in the GBM dataset. Box plots comparing the expression levels of 9 selected genes in GBM samples (blue) and control tissues (green). Differential expression was assessed using a log2 fold change (Log2FC) cutoff of 1 and a p-value threshold of 0.01. Statistical significance was determined through one-way ANOVA.

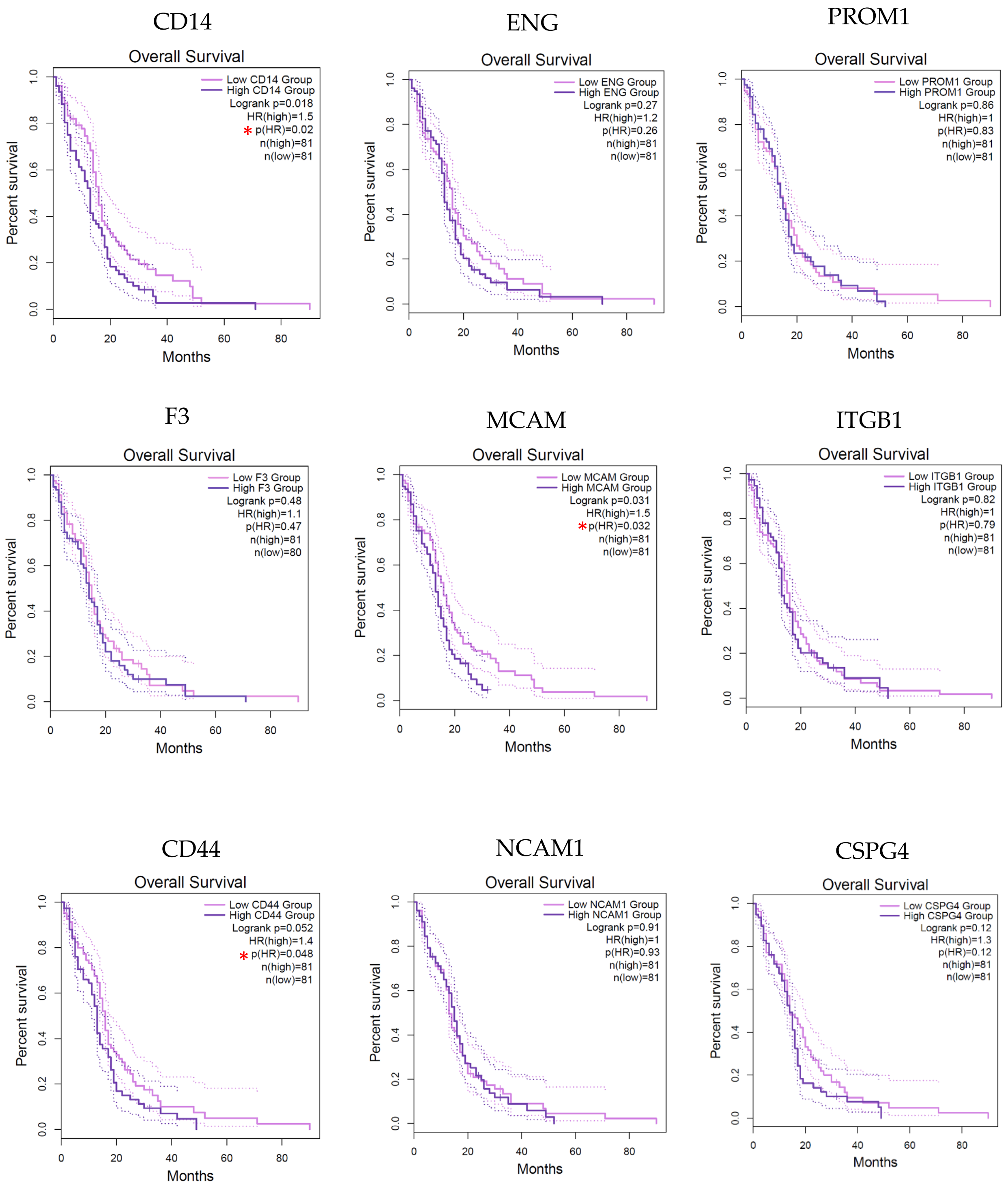

Supplementary Figure S4. Overall survival (OS) analysis. Kaplan-Meier curves showing gene expression levels for each of the nine EVs markers, analyzed using the log-rank test (Mantel-Cox test) for hypothesis testing. The group cutoff for both high and low expression was set at the top and bottom 25%, respectively. The analyses were performed using GEPIA2 tool, leveraging data from The Cancer Genome Atlas (TCGA) for expression analysis of 163 GBM samples and the Genotype-Tissue Expression (GTEx) database for comparison with 207 control cortex samples.

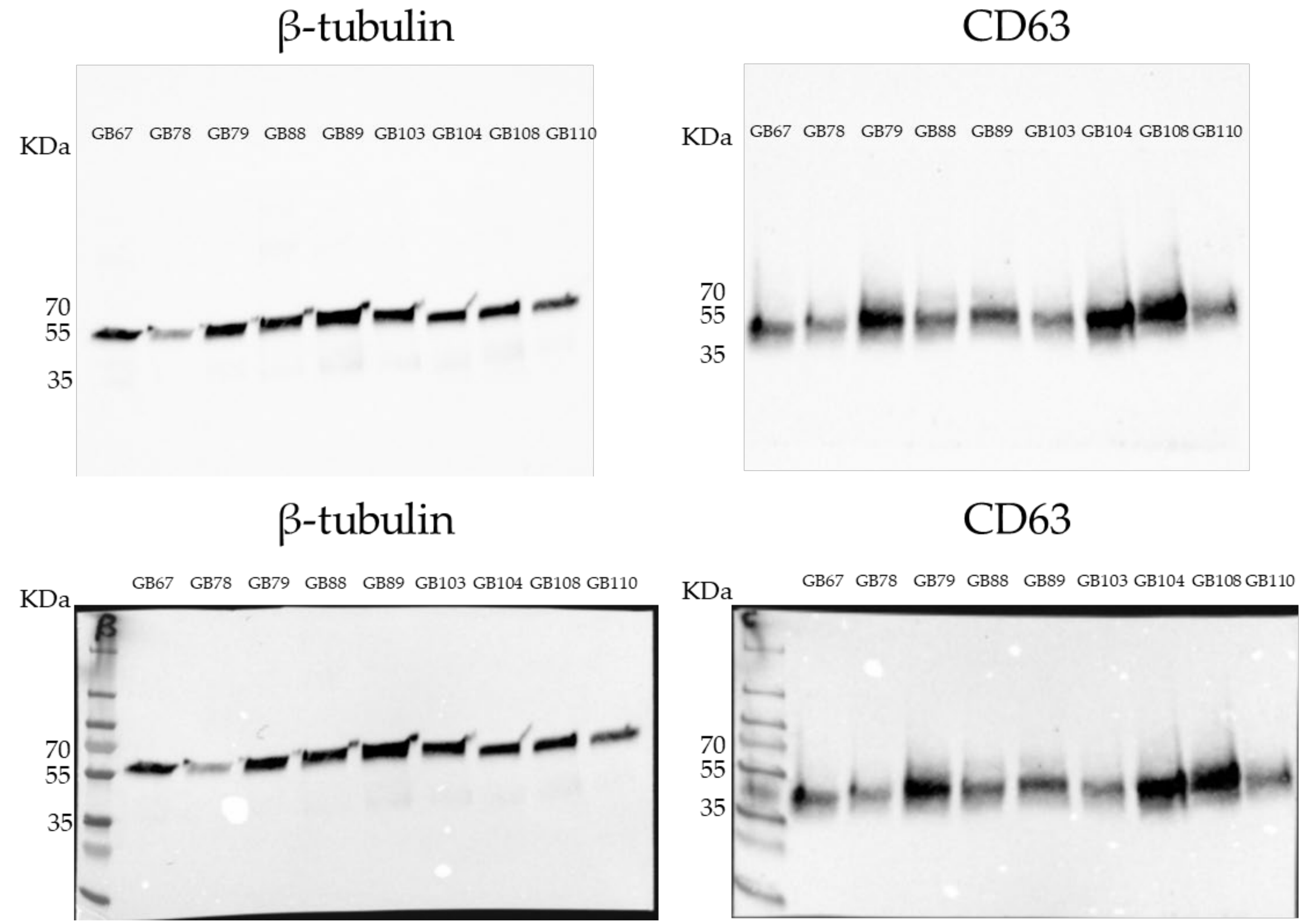

Supplementary Figure S5. Uncropped western blot images without (top image) and merged with the colorimetric image showing the reference ladder bands for molecular weight in KDa (bottom image).

β-tubulin

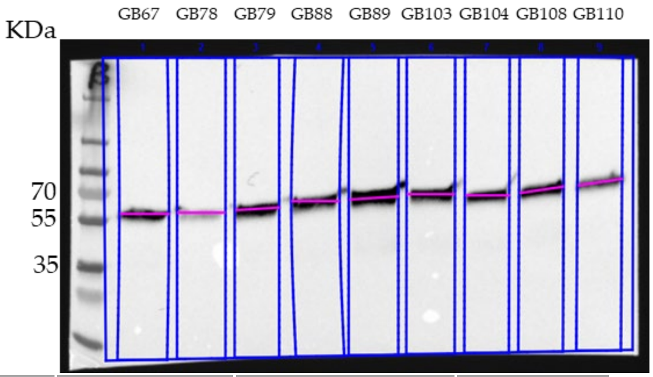

Lane	Relative Front	Adj. Volume (Int)	Volume (Int)
1	0,516949	9468147	11595090
2	0,514894	3548744	5179742
3	0,504274	10900890	12979400
4	0,480687	11217144	14009232
5	0,472103	11161137	13251186
6	0,461207	12017044	14671458
7	0,467532	9192260	11180400
8	0,450216	10360735	12317690
9	0,426087	7214445	8980055

CD63

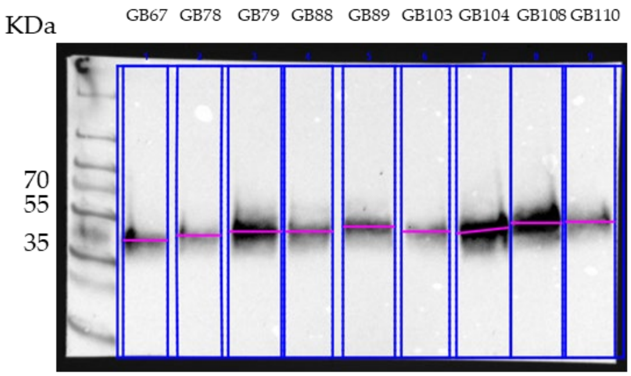

Lane	Relative Front	Adj. Volume (Int)	Volume (Int)
1	0,597458	13895280	17782128
2	0,580508	8829144	12099672
3	0,567797	28781147	34485140
4	0,567797	13081068	17075448
5	0,550847	14993328	20337366
6	0,567797	9091602	12988482
7	0,563559	33648164	40868696
8	0,538136	33883206	40184760
9	0,533898	13449120	17048120

Supplementary Figure S6. Densitometry readings/intensity ratio of each band performed using Image Lab 6.0.1 (Bio-Rad).
